# Chemical composition, antioxidant activities, and enzyme inhibitory effects of *Lespedeza bicolour* Turcz. essential oil

**DOI:** 10.1080/14756366.2025.2460053

**Published:** 2025-02-06

**Authors:** Jiadong Zhu, Ziyue Xu, Xu Liu

**Affiliations:** ^a^ SDU‐ANU Joint Science College, Shandong University, Weihai, China; ^b^ Department of Ocean Science, The Hong Kong University of Science and Technology, Kowloon, China; ^c^ Marine College, Shandong University, Weihai, China

**Keywords:** *Lespedeza bicolour* Turcz., essential oil, chemical composition, bioactivities, molecular docking

## Abstract

*Lespedeza bicolour* Turcz. is a traditional medicinal plant with a wide range of ethnomedicinal values. The main components of *L. bicolour* essential oil (EO) were β-pinene (15.41%), β-phellandrene (12.43%), and caryophyllene (7.79%). The EO of *L. bicolour* showed antioxidant activity against ABTS radical and DPPH radical with an IC_50_ value of 0.69 ± 0.03 mg/mL and 10.44 ± 2.09 mg/mL, respectively. The FRAP antioxidant value was 81.96 ± 6.17 μmol/g. The EO had activities against acetylcholinesterase, α-glucosidase, and β-lactamase with IC_50_ values of 309.30 ± 11.16 μg/mL, 360.47 ± 35.67 μg/mL, and 27.54 ± 1.21 μg/mL, respectively. Molecular docking showed methyl dehydroabietate docked well with all tested enzymes. Sclareol and (+)-borneol acetate showed the strongest binding affinity to α-glucosidase and β-lactamase, respectively. The present study provides a direction for searching enzyme inhibitors for three tested enzymes and shows *L. bicolour* EO possesses the potential to treat a series of diseases.

## Graphical abstract

**Figure:**
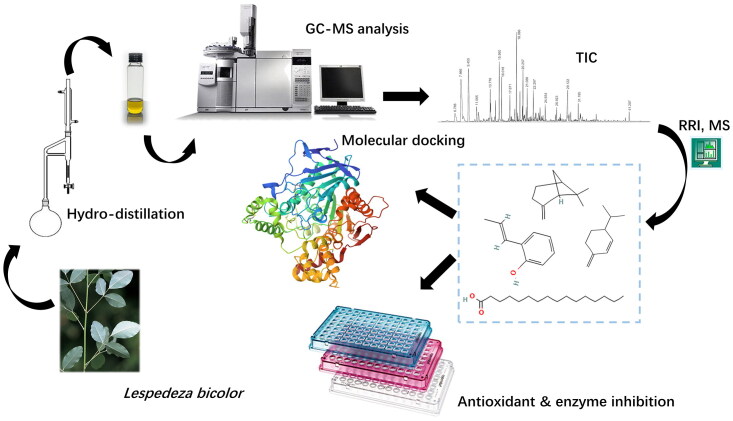

## Introduction

In recent years, the prevalence of diseases associated with oxidative stress has raised significant global health concerns^1^. Oxidative stress, characterised by the excessive generation of reactive oxygen species (ROS), is pivotal in various pathological conditions^2^. The main types of ROS include superoxide, hydroperoxyl radical, singlet radical, and hydroxyl radical^3^. Free radicals exist independently and contain an unpaired electron in their atomic orbital. These radicals can interact with various constituents within tissues, exert effects, and participate in diverse physiological processes such as redox regulation, mitogenic responses, and cellular signalling pathways^4^. However, the overabundance of free radicals can disrupt the organism’s homeostasis, leading to acute and chronic dysfunctions, including conditions such as Alzheimer’s disease (AD)^5^ and diabetes mellitus (DM)^6^.

AD is a progressive neurodegenerative disorder distinguished by the premature extracellular accumulation of diffuse and neuritic plaques (constituted of Aβ peptides) and subsequently by the intracellular emergence of neurofibrillary tangles (comprising hyperphosphorylated tau protein) within the brain^7^. It is worth noting that oxidative stress, induced by factors such as immune response and inflammation, stands as a primary culprit for Aβ plaque formation. Therefore, managing oxidative stress through mitigating the overproduction of ROS in AD patients presents a promising therapeutic strategy, wherein plant-derived antioxidants can serve as a pivotal component^8^. Another factor is the overactivity of the enzyme acetylcholinesterase (AChE), which quickly hydrolyses acetylcholine (ACh) to yield choline and acetate anions, thereby curtailing the prolonged excitation induced by neurotransmitters upon the postsynaptic membrane. This overactivity leads to the reduction of ACh levels and cholinergic dysfunction, eventually contributing to the cognitive deficits observed in AD patients^9^. Therefore, natural compounds exhibiting both antioxidant activities and AChE inhibitory effects hold great promise in treating AD.

DM is a complex metabolic disorder characterised by high blood glucose levels resulting from pancreatic β-cell destruction, peripheral insulin resistance, and compensatory insulin hypersecretion from pancreatic islets^10^. Oxidative stress is closely linked to DM. ROS can directly oxidise particular proteins implicated in the diabetic pathway, thereby inducing redox modifications in these proteins^11^. Moreover, hyperglycaemia is involved in initiating vascular complications of DM, while OS heightened ROS production, playing a pivotal role in their pathogenesis^12^. The development of diabetes is also associated with the overactivity of the enzyme α-glucosidase, which is present at the brush border of the intestine and plays an important role in carbohydrate digestion to form mono-, di-, and polysaccharides^13^. Hence, therapies targeting antioxidative effects and α-glucosidase inhibition have arisen as prospective strategies, providing insights into treating DM.

Due to the long-term use of antibiotics, bacterial drug resistance has become a problem demanding prompt solutions. β-lactam antibiotic, one of the most important weapons to treat bacterial infections, is challenged by bacterial resistance^14^. Among the resistance mechanisms, the expression of β-lactamase enzymes is a subject of extensive research. The combined use of β-lactamase inhibitors with broad-spectrum activity β-lactam antibiotics has successfully addressed bacterial resistance^15^. Therefore, the addition of β-lactamase inhibitors in β-lactam antibiotics has arisen as a prospective strategy, providing insights into solving bacterial drug resistance problem^16^.

Currently, various synthetic drugs have been employed to manage the diseases mentioned above. However, some have shown side effects^17^. To solve this problem, an increasing number of researchers shift towards “nature”, as naturally-occurring components exhibit advantageous alternatives for advancing novel therapies. Plants, especially, have played a crucial role in drug discovery research^18^. Essential oil (EO), a complex mixture of volatile, lipophilic compounds that are typically derived from plant secondary metabolism, is characterised by their distinct aroma and flavour and has been shown to possess a wide range of bioactivities, including antimicrobial, antioxidant, anti-inflammatory, and enzyme inhibitory properties^19^. EO has been used for centuries for therapeutic purposes since antiquity and has more recently gained attention as a potential source of new drugs and therapeutic agents^20^. Due to the fact that EO is a mixture of bio-organic small molecules, they exhibit broad-spectrum activity in areas such as enzyme inhibition, disease treatment, and antimicrobial activity. The molecular diversity allows for extensive screening in relevant fields, providing opportunities to discover potential therapeutic agents^21^. Given the broad-spectrum effects of EO, there is great potential in discovering new therapeutic agents through comprehensive screening of EO’s bioactivities in relevant domains, coupled with molecular docking analysis.

*Lespedeza*, the genus belonging to the Fabaceae family, consists of 40 species distributed in East Asia and eastern North America and introduced from Malesia to Australia. The plants of the genus are herbaceous or shrubby and have cleistogamous and chasmogamous flowers^22^^,^^23^, and have been used in traditional medicine to treat various diseases, such as nephritis, azotaemia, inflammation, hyperpigmentation, diabetes, and diuresis^24^. Recent studies have also shown effectiveness in treating cough and fever, and they have oestrogenic effects and inhibitory effects on xanthine oxidase and tyrosinase^25^. Therefore, *Lespedeza* species hold promise as alternative therapies for various diseases, with their natural products potentially serving as enzyme inhibitors of key targets of diseases. *Lespedeza bicolour* Turcz., is an erect shrub with multiple branches and compound leaves consisting of three leaflets. It has lanceolate and linear stipules with two present per leaf and typically produces conical panicle inflorescences. *L. bicolour* holds great importance in economics because lots of derivatives extracted from *L. bicolour* hold several important bioactivities. Researchers have successfully isolated several bioactive compounds from *L. bicolour*, including dimeric flavonoid lespebicolin B, polyphenolic compound pterocarpans lespedezol, coumestans, and arylbenzofurans. These compounds have been found to exhibit a range of beneficial properties, such as antioxidant, cytotoxic, anti-HSV-1, and antiproliferative activities^26–28^. In addition, methanolic crude extracts derived from *L. bicolour* have demonstrated potential anti-inflammatory, antioxidant, anticancer, and depigmentation effects, highlighting the plant’s potential for further drug development^29^^,^^30^.

Historical pharmacopeias and contemporary literature document the broad biological activities and high medicinal value of *L. bicolour*. However, no previous studies have investigated the chemical composition and bioactivities of *L. bicolour* EO, representing a significant knowledge gap in this repository of bioactive compounds. To broaden the molecular database of *L. bicolour*, with the aim of uncovering traditional medicinal resources as potential therapies, we conducted exploratory research on the EO from *L. bicolour*, including chemical composition, antioxidant activity, as well as its inhibitory effects on AChE, α-glucosidase, and β-lactamase. Furthermore, molecular docking was employed to validate the mechanisms underlying the enzyme-inhibitory effects of *L. bicolour* EO.

## Materials and methods

### Plant material

The plant sample was gathered from Shandong University (122.054903 E, 37.537827 N), Weihai City, Shandong Province, China, in September 2023. The species was identified as *Lespedeza bicolour* Turcz. by Prof. Hong Zhao and deposited in the Centre for Bioscience Analysis and Test, Shandong University, Weihai, China, with registration number EO2310. Before EO distillation, the plant material was maintained under refrigeration (-18 °C). This species is not listed in China’s National Key Protected Wild Plants, China’s Red List of Biodiversity, List of Very Small Population (Narrowly Distributed) Protected Species, and the Appendix to the Convention on International Trade in Endangered Species of Fauna and Flora (CITES). The collection has obtained official permission from the Weihai Hi-tech Industrial Development Zone Agricultural Development Bureau.

### EO extraction

The dry leaves and stems of the plant materials (500 grams) were crushed into small pieces, mingled with 3 L ultrapure water in a 5 L round bottom flask, using a Clevenger-type apparatus to hydrodistill for approximately 4.0 h. The EO was then separated from the aqueous layer using diethyl ether. Anhydrous Na_2_SO_4_ was used to dry the isolated EOs, and samples were stored at 4 °C in an amber phial before the analysis.

### GC-MS and GC-FID analysis

Gas chromatography-mass spectrometer (GC-MS) and gas chromatography-flame ionisation detector (GC-FID) methods were used to determine the EO components and their content^31^^,^^32^. GC-MS analysis was carried out using an Agilent 7890–5975 C gas chromatograph–mass spectrometer equipped with a fused silica capillary column type HP-5MS (30 m × 0.25 mm × 0.25 µm, Agilent, Santa Clara, CA, USA). The interface and injector temperatures were set at 280 °C and 260 °C, respectively. The oven temperature was initially 50 °C. This was maintained for 4 min, programmed from 50 °C to 280 °C at a 6 °C/min rate, and held steady for 3 min. Helium was used as the carrier gas at a 1.0 ml/min velocity. The mass spectrometer conditions were as follows: electron impact (EI) mode (electron energy = 70 eV), scan range of 25–500 amu, scan rate of 4.0 scans/s, and quadrupole temperature of 150 °C. A 1% w/v sample solution in n-hexane was prepared, and 0.3 μL was injected using a splitless mode.

The GC-FID analysis was conducted using a PerkinElmer gas chromatograph (Clarus 500, Shelton, CT, USA) equipped with an HP-5 column (30 m × 0.25 mm with film thickness 0.25 microns, Agilent Technologies, USA). The injector temperature was 260 °C, and the detector temperature was 305 °C. The oven temperature was initially 50 °C, was maintained for 4 min, and then programmed from 50 °C to 280 °C at the rate of 6 °C/min and held steady for 3 min. Helium was used as the carrier gas at a 1.0 ml/min velocity.

The components of the EO were identified using the Agilent MassHunter Qualitative Analysis software (v 10.0). Mass spectrometric data and Kovats retention indices (RIs) were compared against those sourced from the commercial libraries (NIST 23 and Adams)^33^. The Kovats RIs were computed using *n*-alkanes (C_8_–C_30_). The relative abundance of components was also ascertained through peak area normalisation^34^.

### Antioxidant activities evaluation

#### ABTS method

The experimental procedure is derived from the previous methods with some modifications^35^^,^^36^. A mixture of 7.0 mM 2,2-azinobis(3-ethylbenzothiazoline-6-sulfonate) (ABTS) solution and 2.6 mM potassium persulfate was prepared to generate the ABTS^•+^ free radical. The mixture was subsequently stored in the dark at room temperature for 12 h, facilitating the entire generation of the ABTS^•+^ free radical^37^. Additionally, ethanolic EO solutions were prepared at 50, 25, 10, 5, 2.5, and 1 mg/mL concentrations. A microplate reader (Epoch, Biotech company, Minneapolis, MN, USA) was utilised to ascertain the absorbance of the solution, delineated as the absorbance at 734 nm six minutes post-initiation of the reaction. For comparison, ethanol was used as the blank and gradient-diluted solvent. Meanwhile, 0.5 mg/mL 6‐hydroxy‐2,5,7,8‐tetramethylchroman‐2‐carboxylic acid (Trolox) was utilised as the positive control. The ABTS radical scavenging capacity (RSC%) was calculated using the Equation (1).

(1) RSC%=A0−AA0×100%

where A_0_ and A are the absorbance of 200 μL diluted ABTS^•+^ solution mixed with 50 μL ethanol and 50 μL sample solution, respectively, at 734 nm. IC_50_ was then calculated. All tests were performed in triplicates.

#### DPPH method

The experimental procedure was adapted from previous research with minor modifications^38^. The concentrations of Trolox (positive control) and the mother solution of the essential oil EO were 1.0 mg/mL and 20 mg/mL, respectively, in an ethanolic system. 50 μL of the positive control or EO solution was pipetted into a 96‐well microplate and 200 μL of a 2,2-diphenyl-1-picrylhydrazyl (DPPH) solution. The microplate was incubated in the dark at 25 °C for 30 min, and then the absorbance was measured at 517 nm using an Epoch microplate absorbance spectrophotometer (Epoch, Biotech company, Minneapolis, MN, USA). The DPPH radical scavenging capacity (RSC%) was calculated using the Equation (2). All tests were performed in triplicates, and the results were averaged.

(2) RSC%=(1−ASample−ASample BlankAControl)×100%

where, *A_Sample_* is the absorbance of the tested sample at different concentrations, *A_Control_* is the absorbance of the control (ethanolic DPPH solution), and *A_Sample Blank_* is the absorbance of the ethanol sample without DPPH.

#### Ferric-Reducing antioxidant power (FRAP) method

This assay was conducted based on previous research^39^^,^^40^. Three stock solutions: (i) pH 3.6 acetate buffer solution, (ii) 10 mmol/L 2,4,6-tripyridyl-s-triazine solution (TPTZ), and (iii) 20 mmol/L Fe^3+^ solution were mixed in a ratio of 10:1:1 and then further diluted 50 times with ethanol to generate the FRAP working solution. Trolox was used to construct the standard curve. The EO samples were gradient-diluted into 5000, 2500, 1000, 500, 250, 100, 50, and 25 μg/mL. The EO samples and FRAP working solution were amalgamated for reaction at 37 °C in dark conditions. To obtain the antioxidant capacity value (Trolox equivalent antioxidant concentration), we bring the absorbance at 593 nm of the sample at the determined concentration back to the standard curve equation. Tests were replicated three times, and the average value of the three tests was taken as the final result.

### Anti-acetylcholinesterase activity test

The inhibitory effect of acetylcholinesterase was measured in a potassium phosphate buffer (PBS) system according to the previously described method with minor modifications^41^. Ethanolic EO solutions (50 mg/mL) were diluted to 2.5, 1, 0.5, 0.25, 0.1, and 0.05 mg/mL by pH 8.0 PBS. Besides, galantamine was used as the positive control. 145 μL of 0.1 mM, pH 8.0 PBS, 20 μL of ethanolic EO solutions, and 15 μL of AChE solution encompassing 0.28 U/mL were mixed in a microplate then refrigerated at 4.0 °C for 20 min. Afterward, add 10.0 μL of 15 mM Acetylthiocholine iodide (ATCI) and 10.0 μL of 2 mM 5,50-Dithiobis-(2-nitrobenzoic acid) (DTNB) homogenising for 60 s. Subsequently, the absorbances were measured at 412 nm for 6 min at 60-s intervals by a microplate reader (Epoch, Biotech company, Minneapolis, MN, USA). All tests were performed in triplicates, and the results were averaged. Equation (3) expresses the acetylcholinesterase inhibition rate:

(3) inhibition%=(KE−KS)KE×100%

where *K_E_* represents the initial reaction rate of the acetylcholinesterase under inhibited conditions; it was calculated using linear regression with absorbance as the dependent variable and time as the independent variable. *K_S_* represents the initial reaction rate of the acetylcholinesterase using the same method but replacing 20 μL of the sample with 20 μL of solution. IC_50_ was calculated using nonlinear regression by GraphPad Prism 10.1.

### Anti-α-glucosidase capacity test

The test was conducted according to previous research^42^ with some modifications to determine the anti-α-glucosidase capacity of EO. Ethanolic EO solutions (25 mg/mL) were diluted to 3.0, 1.75, 1, 0.75, 0.50, 0.25, and 0.125 mg/mL by pH 7.0 PBS. Besides, ethanolic acarbose was diluted from 1 mg/mL to concentrations of 1000, 500, 250, 175, 100, 75, 50, and 25 ng/mL as positive controls by pH 7.0 PBS. A microplate reader (Biotek, USA) was used to determine the absorbance of the solutions. 20 μL of sample, 80 μL of 100 mM, pH 6.8 PBS, and 40 μL of α-glucosidase solution encompassing 0.25 U/mL were combined in a microplate and then incubated at 30 °C for 10 min. Afterward, 20 μL of 3.0 mg/mL 4-Nitrophenyl-β-D- glucopyranoside (pNPG) was added, and the mixture was homogenised for 60 s, then incubated at 30 °C for 4 min. Subsequently, the absorbances were assessed at 410 nm for 6 min at 60-s intervals. All tests were performed in triplicates, and the results were averaged. The α-glucosidase inhibition rate is expressed using the Equation (4).

(4) $$\begin{array}{c} i\text{nhibition}\%=\frac{\left(K_{E}-K_{S}\right)}{K_{E}}\times 100\% \end{array}$$

where *K_E_* represents the initial reaction rate of the α-glucosidase under inhibited conditions and is calculated using linear regression with absorbance as the dependent variable and time as the independent variable. *K_S_* represents the initial reaction rate of the glucosidase using the same method but substituting the sample with the solution. IC_50_ was calculated using nonlinear regression by GraphPad Prism 10.1.

### Test for β-lactamase inhibitory effect

The β-lactamase inhibitory effect was assessed using a previously described method with some modifications^43^. Ethanolic EO solutions (50 mg/mL) were diluted to concentrations of 75, 37.5, 25, 17.5, 10, 5, and 2.5 μg/mL by pH 7.0 PBS. Besides, the Clavulanate Potassium solution was used as the positive control. Then, in each microplate well, 20 μL of sample, 30 μL of 50 mM, pH 7.0 PBS, and 100 μL of β-lactamase solution at 1000 U/mL were mixed and incubated at 30 °C for 10 min. Subsequently, 50 μL of 0.1 mg/mL Nitrocefin was added and then incubated at 30 °C for another 10 min. The absorbance was measured at 489 nm. 100 μL β-lactamase solution was used to replace 100 μL PBS solution as a blank reaction. An enzymatic activity determination was performed using a 20 μL PBS solution instead of a 20 μL EO sample. Next, 130 μL PBS was used to replace 100 μL β-lactamase solution as the sample blank reaction. All tests were performed in triplicates, and the results were averaged. The β-lactamase inhibition rate is calculated using the Equation (5):

(5) $$\begin{array}{c} \text{inhibition}\%=\frac{1-\left(A_{S}-A_{\text{sb}}\right)}{\left(A_{E}-A_{B}\right)}\times 100\% \end{array}$$

where *A_S_* is the absorbance of the EO-containing sample, *A_sb_* is the absorbance of the sample blank reaction, *A_E_* absorbance of the reaction in which the enzyme was not inhibited, and *A_B_* is the absorbance of the blank reaction. IC_50_ was assessed using nonlinear regression by GraphPad Prism 10.1.

### Molecular docking

Complexes of AChE (*Tetronarce californica*, PDB code: 1EA5)^44^, α-glucosidase (*Saccharomyces cerevisiae*, PDB code: 3AJ7)^45^, and β-lactamase (*Enterobacter cloacae,* PDB code: 7TI1)^46^ were obtained from the PDB database. PyMol v2.2.0 was utilised to remove the ligands and crystal water, thus obtaining receptor structures. Hydrogen atoms were added to the receptor structures to ensure the correct protonation states. The 3D structures of components in *L. bicolour* EO used in the docking experiments were gathered from the CAS SciFinder Discovery Platform (https://scifinder-n.cas.org/) with Chem3D to minimise molecule energy. These components were selected as ligands to interact with the enzyme receptors.

Autodock Vina 1.5.7 software^47^ was used for semi-flexible molecular docking. As the previous study suggested^48^, in order to ensure the accuracy of our docking results and the efficiency of the experiment, the value of exhaustiveness was set at 25, and maximum geometric shapes were set as 20, with the remaining parameters set to default values. The active site of each receptor (AChE (x, y, z) = (2.50, 65.72, 66.39); α-glucosidase (x, y, z) = (17.93, −8.62, 19.84); β-lactamase (x, y, z) = (52.03, 11.33, 54.1158)) was detected by PrankWeb (https://prankweb.cz/)^49^. The docking box was set at the active centre and included the entire active pocket. The binding energies and the interaction between the ligand and proteins were evaluated in the docking process. Discovery Studio visualiser and Pymol v2.2.0 were used to visualise the docking results.

## Result and discussion

### EO yield, chemical compositions Analysis

0.10 ml EO was hydrodistilled from 0.50 kg *L. bicolour* biomass. Several factors can affect the yield of EO, such as different extraction methods^50^, distillation time^51^, and drying methods^52^. Besides, the extracted EO may exhibit variances in yield and composition due to differences in climate, soil composition, plant organ, age, and biosynthesis processes^53^. Phylogenetically closely related plants often manifest similarities in their secondary metabolic pathways, leading to similar EO yields. In Fabaceae, compared with the yield of *Dalea mutisii* Kunth’s EO (0.25% w/w)^54^, the yield of *L. bicolour*’s EO is 0.20% (v/w), which is slightly lower, showing similar EO yields within the same family. In contrast, when compared to industrially produced essential oils such as those of *Ocimum basilicum* and *Citrus reticulata*, which yield 1.70% and 0.30%, respectively^55^^,^^56^, the yield of *L. bicolour* essential oil is relatively low, which presents challenges for industrial applications. Nonetheless, it opens up the inspiration for how variations in chemical mixtures contribute to the different biological activities.

**Figure 1.**
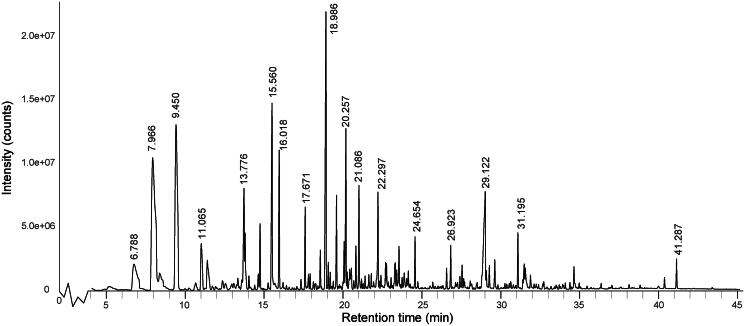
The total ion chromatogram of *L. bicolour* EO derived from GC–MS data.

The total ion chromatogram (TIC) of *L. bicolour* is shown in Figure 1. Eighty-three compounds were identified, representing 98.44% of the EO composition. The identified components are listed in Table 1 with their retention time (RT), retention index (RI), relative peak area, identification method, and CAS ID. The main components are β-pinene (15.41%), β-phellandrene (12.43%), caryophyllene (7.79%), palmitinic acid (5.49%), 2-propenylphenol (4.94%) and α-pinene (4.01%). Terpenoids comprise 66.50% of the identified compounds, followed by aliphatic compounds (18.72%), aromatic compounds (10.03%), and others. The high proportion of twenty-two identified terpenes suggests a specific terpene chemotype in *L. bicolour.* Compared with *Dalea mutisii* Kunth’s EO, the compositions of the *L. bicolour* EO are similarly abundant in β-pinene, and β-phellandrene, but lack α-pinene^54^.

**Table 1.** Chemical composition of EO distilled from *Lespedeza bicolour* Turcz.

No.	RT	Compound	RI^calc^	RI^lib^	Area (%)	Identification method	CAS ID
1	6.788	α-Pinene	934	937	4.01%	RRI, MS	80-56-8
2	7.966	β-Pinene	977	974	15.41%	RRI, MS	127-91-3
3	8.402	β-Myrcene	992	991	1.23%	RRI, MS	123-35-3
4	9.450	β-Phellandrene	1030	1031	12.43%	RRI, MS	555-10-2
5	10.694	Octanol	1076	1070	0.44%	RRI, MS	111-87-5
6	11.065	Terpinolene	1090	1088	2.28%	RRI, MS	586-62-9
7	11.444	Linalool	1104	1099	1.76%	RRI, MS	78-70-6
8	12.401	L-Pinocarveol	1141	1139	0.42%	RRI, MS	547-61-5
9	12.608	Camphene hydrate	1149	1148	0.37%	RRI, MS	465-31-6
10	13.001	Pinocarvone	1165	1162	0.34%	RRI, MS	30460-92-5
11	13.116	Camphol	1169	1167	0.32%	RRI, MS	507-70-0
12	13.378	Terpene-4-ol	1179	1177	0.38%	RRI, MS	562-74-3
13	13.639	Cryptone	1190	1184	0.37%	RRI, MS	500-02-7
14	13.776	L-α-Terpineol	1195	1190	3.01%	RRI, MS	10482-56-1
15	13.841	Methyl salicylate	1198	1192	1.65%	RRI, MS	119-36-8
16	14.087	Decanal	1208	1206	0.33%	RRI, MS	112-31-2
17	14.681	Citronellol	1233	1228	0.40%	RRI, MS	106-22-9
18	14.796	Methylthymol	1238	1235	1.37%	RRI, MS	1076-56-8
19	15.309	Geraniol	1260	1255	0.19%	RRI, MS	106-24-1
20	15.560	2-Propenylphenol	1270	1264	4.94%	RRI, MS	23619-59-2
21	16.018	(+)-Borneol acetate	1290	1293	3.04%	RRI, MS	20347-65-3
22	16.258	Theaspirane	1300	1302	0.15%	RRI, MS	36431-72-8
23	17.398	α-Cubebene	1351	1351	0.24%	RRI, MS	17699-14-8
24	17.671	Eugenol	1364	1357	1.64%	RRI, MS	97-53-0
25	17.878	Ylangene	1373	1372	0.36%	RRI, MS	14912-44-8
26	17.982	Copaene	1378	1376	0.30%	RRI, MS	3856-25-5
27	18.189	Geranyl acetate	1387	1382	0.19%	RRI, MS	105-87-3
28	18.298	β-Cubebene	1392	1389	0.10%	RRI, MS	13744-15-5
29	18.631	Longifolene	1408	1406	0.75%	RRI, MS	475-20-7
30	18.986	Caryophyllene	1425	1419	7.79%	RRI, MS	87-44-5
31	19.133	β-Copaene	1432	1432	0.42%	RRI, MS	18252-44-3
32	19.258	trans-α-Bergamotene	1438	1435	0.29%	RRI, MS	13474-59-4
33	19.455	Isogermacrene D	1447	1448	0.15%	RRI, MS	317819-80-0
34	19.662	Humulene	1457	1454	1.81%	RRI, MS	6753-98-6
35	20.077	Cadina-1(6),4-diene	1477	1474	0.11%	RRI, MS	20085-11-4
36	20.148	γ-Muurolene	1481	1477	0.95%	RRI, MS	30021-74-0
37	20.257	Germacrene D	1486	1481	3.38%	RRI, MS	23986-74-5
38	20.339	β-Ionone	1490	1491	0.27%	RRI, MS	14901-07-6
39	20.453	1,11-Oxidocalamenene	1496	1493	0.12%	RRI, MS	143785-42-6
40	20.502	γ-Amorphene	1498	1496	0.45%	RRI, MS	6980-46-7
41	20.606	α-Muurolene	1503	1499	0.57%	RRI, MS	10208-80-7
42	20.753	(±)-Valencene	1511	1507	0.15%	RRI, MS	24741-64-8
43	20.895	γ-Cadinene	1518	1513	0.87%	RRI, MS	39029-41-9
44	21.086	δ-Cadinene	1528	1524	1.95%	RRI, MS	483-76-1
45	21.25	Cadina-1,4-diene	1536	1533	0.13%	RRI, MS	16728-99-7
46	21.479	α-Calacorene	1548	1542	0.18%	RRI, MS	21391-99-1
47	21.872	Nerolidol	1568	1564	0.20%	RRI, MS	7212-44-4
48	22.194	(-)-Spathulenol	1584	1577	0.12%	RRI, MS	77171-55-2
49	22.297	Caryophyllene oxide	1589	1581	1.98%	RRI, MS	1139-30-6
50	22.379	Longifolenaldehyde	1594	1591	0.08%	RRI, MS	19890-84-7
51	22.483	Mintketone	1599	1595	0.19%	RRI, MS	73809-82-2
52	22.777	Humulene epoxide 2	1615	1606	0.43%	RRI, MS	19888-34-7
53	22.843	6-Methyl-2-(4-methylcyclohex-3-en-1-yl)hepta-1,5-dien-4-ol	1618	1608	0.41%	RRI, MS	38142-56-2
54	23.132	Epicubenol	1634	1627	0.17%	RRI, MS	19912-67-5
55	23.394	T-Muurolol	1648	1642	0.73%	RRI, MS	5937-11-1
56	23.492	δ-Cadinol	1653	1645	0.28%	RRI, MS	19435-97-3
57	23.634	α-Cadinol	1661	1653	0.70%	RRI, MS	481-34-5
58	23.966	(*E*)-Tetradec-2-enal	1679	1673	0.46%	RRI, MS	51534-36-2
59	24.125	Eudesma-4(15),7-dien-1β -ol	1687	1688	0.12%	RRI, MS	119120-23-9
60	24.228	4(15),5,10(14)-Germacratrien-1-ol	1693	1690	0.22%	RRI, MS	81968-62-9
61	24.654	1-Pentadecanal	1716	1715	0.93%	RRI, MS	2765-11-9
62	24.828	(*E*)-Farnesol	1726	1722	0.15%	RRI, MS	106-28-5
63	25.783	1-Pentadecanol	1780	1778	0.14%	RRI, MS	629-76-5
64	26.923	Perhydrofarnesyl acetone	1847	1844	0.79%	RRI, MS	502-69-2
65	27.229	Platambin	1865	1867	0.16%	RRI, MS	58556-80-2
66	27.507	1-Hexadecanol	1882	1880	0.22%	RRI, MS	36653-82-4
67	27.649	(8*Z,*11*Z*)-Heptadecadienal	1890	1886	0.40%	RRI, MS	56797-42-3
68	27.747	Methyl 4,7,10-hexadecatrienoate	1896	1892	0.15%	RRI, MS	17364-31-7
69	28.167	Farnesyl acetone	1922	1918	0.16%	RRI, MS	1117-52-8
70	28.604	Isophytol	1949	1948	0.11%	RRI, MS	505-32-8
71	29.122	Palmitinic acid	1981	1968	5.49%	MS	57-10-3
72	29.384	Manoyl oxide	1997	1991	0.40%	RRI, MS	596-84-9
73	29.727	Epimanoyl oxide	2019	2011	0.62%	RRI, MS	1227-93-6
74	30.737	Stearol	2085	2082	0.18%	RRI, MS	112-92-5
75	31.195	Phytol	2115	2114	1.09%	RRI, MS	150-86-7
76	31.599	Methyl stearolate	2143	2141	0.68%	RRI, MS	1120-32-7
77	31.675	9-Octadecenoic acid	2148	2144	0.73%	RRI, MS	2027-47-6
78	31.997	Octadecanoic acid	2170	2172	0.27%	RRI, MS	57-11-4
79	32.842	Sclareol	2228	2227	0.15%	RRI, MS	515-03-7
80	34.506	Methyl dehydroabietate	2348	2341	0.13%	RRI, MS	1235-74-1
81	34.763	Octadecanamide	2367	2374	0.60%	RRI, MS	124-26-5
82	40.524	Squalene	2831	2827	0.21%	RRI, MS	111-02-4
83	41.287	Nonacosane	2899	2900	0.58%	RRI, MS	630-03-5
	Terpenoids				66.50%		
	Aromatic compounds				10.03%		
	Aliphatic compounds				18.72%		
	Other compounds				3.19%		
	Total identified				98.44%		

Concentration was calculated from the total ion chromatogram; RI^Calc^: Calculated retention index on an HP-5MS column; RI^lib^: Retention index was obtained from the NIST/EPA/NIH 2023 Mass Spectral Database. RRI: Relative retention indices calculated against n-alkanes; Identification method based on the relative retention indices (RRI) of authentic compounds on the HP-5MS column; MS, identified based on computer matching of the mass spectra with NIST/EPA/NIH 2023 Mass Spectral Database and comparison with literature data.

The abundance of terpenoids over aromatic compounds in *L. bicolour* EO can be attributed to the biosynthetic pathways. Typically, the biosynthetic pathways of terpenoids and aromatic compounds are separated in plants, but they may coexist in some cases, with one major pathway taking over^19^. The core terpenoid biosynthetic pathways in plants are primarily the cytosolic mevalonic acid (MVA) pathway and the plastidial methylerythritol phosphate (MEP) pathway, while aromatic compounds centre in general phenylpropanoid pathway^57^^,^^58^. In the case of *L. bicolour* EO, MVA, and MEP pathways are presumably dominant. β-Pinene (15.41%), the primary component found in *L. bicolour* EO, is the well-known representative of a broad family of monoterpenes, exhibiting diverse biological activities such as inhibitory effects on breast cancer and leukaemia^59^ and toxic effects on membranes^60^. Meanwhile, β-pinene was tested, exhibiting antimicrobial activity with a mechanism of damaging these microorganisms’ cellular integrity. Moreover, antibiotic resistance modulation, neuroprotective effects, and antioxidant activities of β-pinene have also been reported^61^. β-Phellandrene (12.43%), a component synthesised from geranyl-diphosphate (GPP) by a nuclear-encoded and plastid localised β-phellandrene synthase (PHLS), has been generally found in various EOs. The EOs abundant in β-phellandrene have been shown to exhibit antibacterial, antioxidant, and nervous system activity^62^ and β-phellandrene was also found to have anti-fungal, anti-inflammatory, anti-hyperalgesic, anti-depressant analgesic, and anticancer activities^63^. Moreover, caryophyllene (7.79%), another prominent antioxidative compound abundant in *L. bicolour* EO, may contribute to the antioxidant potential of *L. bicolour* EO while also exhibiting numerous other biological properties, including anti-inflammatory, anxiolytic, antitumor, and antidepressive effects^64^. These principal components play a role in the biological properties of *L. bicolour* EO. However, some other ingredients in smaller proportions exhibit synergistic effects, while others reveal antagonistic effects. The biological properties of *L. bicolour* EO were determined by the essential oil system rather than specific molecules. Considering the bioactivities mentioned above that *L. bicolour* EO presumably possesses, We evaluated the antioxidant capacity and the inhibitory effects of enzymes that represent key therapeutic targets in diseases. Meanwhile, molecular docking was employed to identify which compounds are more likely to inhibit these enzymes.

### Antioxidant activities analysis

**Figure 2.**
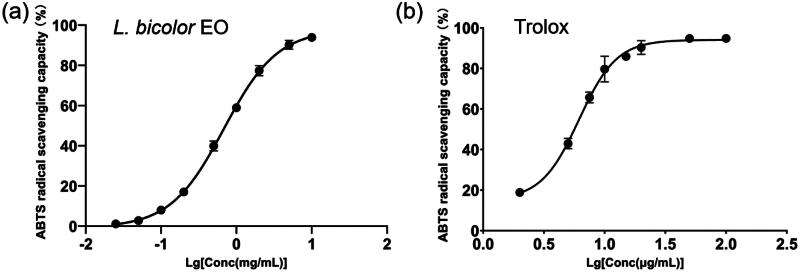
Variation in ABTS radical scavenging percentage with varying concentrations for *L. bicolour* EO (a), Trolox (b).

The results of the ABTS assay are shown in Figure 2. The IC_50_ of ABTS radical scavenging capacity of *L. bicolour* EO and Trolox (positive control) were 0.69 ± 0.03 mg/mL and 6.15 ± 0.15 μg/mL, respectively. Compared with ABTS radical scavenging IC_50_ of EO from another Fabaceae plant, *Tadehagi triquetrum* (IC_50_ = 2.12 ± 0.05 mg/mL)^65^, *L. bicolour* EO shows stronger ABTS scavenging capacity, indicating its potential to be utilised as a natural-derived antioxidant.

**Figure 3.**
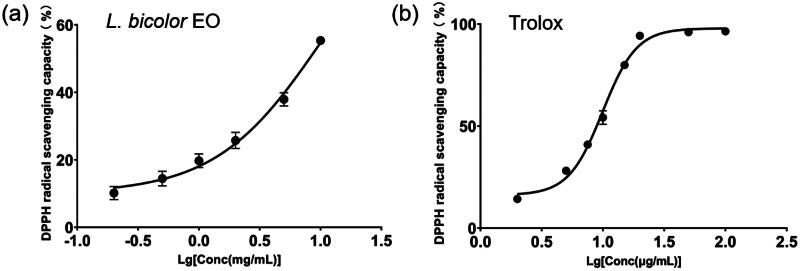
Variation in DPPH radical scavenging percentage with varying concentrations for *L. bicolour* EO (a), Trolox (b).

The results of the DPPH assay are shown in Figure 3. The IC_50_ values of the DPPH scavenging capacity of *L. bicolour* EO and Trolox were 10.44 ± 2.09 mg/mL and 9.94 ± 0.20 μg/mL, respectively. Compared with *Dialium guineense* leaf EO (IC_50_ = 931.7 ± 0.6 μg/mL), *L. bicolour* EO shows weaker DPPH scavenging capacity^66^. The ABTS radical scavenging activity of *L. bicolour* EO was greatly better than the DPPH radical scavenging activity. The different results between the two methods can be attributed to differences in the mechanism, such as the stereoselectivity of the radicals or the solubility of extracts in different testing systems. Both ABTS and DPPH assays obey a “Mixed mode” which includes hydrogen atom transfer, electron transfer, and proton-coupled electron transfer mechanisms. However, DPPH primarily relies upon the hydrogen atom transfer mechanism, while ABTS mainly operates through the electron transfer mechanism^67^^,^^68^. Therefore, it is reasonable to suggest that some major antioxidative compounds in *L. bicolour* EO may primarily terminate free-radical chains through the electron transfer mechanism rather than the hydrogen atom transfer mechanism. For example, α-terpineol (3.01%), which is comparatively abundant in the EO of *L. bicolour*, has been observed to exhibit a heightened scavenging capacity for the ABTS^+•^ free radical as compared to the DPPH^+•^ free radical^69^. α-terpineol contains an alcoholic hydroxyl group, which could produce protons and hydrocarbon oxygen anions during its ionisation equilibrium. After the proton dissociation, a single electron on the oxygen anion is transferred to ABTS^+•^, forming a stable ABTS. The main mechanism underlying the free radical scavenging aptitude of α-terpineol entails a preferential loss of protons, followed by the transfer of a single electron, rather than the hydrogen atom transfer, which is the preferred mode of DPPH^70^. Additionally, the nitrogen atom at the reaction centre of DPPH is surrounded by three phenyl rings, while the reaction centre of ABTS is relatively exposed. The reason mentioned above could explain the relatively lower DPPH scavenging capacity of *L. bicolour* EO.

The FRAP assay was conducted to evaluate the antioxidant activity. In contrast to previous methodologies that focused on free radical scavenging activities, the FRAP assay evaluates the overall antioxidant capacity of the essential oil system. The reaction is non-specific and based on single electron transfer, wherein any half-reaction with a less-positive redox potential under the given reaction conditions than the Fe^III^/Fe^III^-TPTZ half-reaction will promote the reduction of Fe^III^^39^. The EO exhibited a value of 81.96 ± 6.17 μmol/g in the current study. Compared with *Malvastrum Coromandelianum* EO (63.24 ± 4.81 µmol/g)^71^, *L. bicolour* EO is slightly higher. Since Fe^II^ is widely recognised as a highly effective pro-oxidant in the food manufacturing industry, the chelating effects of *L. bicolour* EO could have advantageous impacts in reducing the pro-oxidant activity. Every method has its limitations. Both ABTS and DPPH assays use synthetic radicals, which may not accurately reflect the behaviour of antioxidants in natural biological systems. Results of those tests can vary significantly based on the conditions used, such as the concentration of the ABTS radical and the reaction time. Besides, the ABTS assay does not involve an oxidisable substrate, which measures “radical trapping ability” rather than true antioxidant activity^72^. In the FRAP assay, no radical reaction occurs. Hence, this test indicates some reducing ability, which may not correlate with true antioxidant activity. Therefore, using multiple approaches to assess antioxidant activity can provide a more comprehensive antioxidant profile and make the research more reliable.

The potent antioxidant capacity of *L. bicolour* EO can be attributed to certain distinctive components. (1) Phenolic compounds, including eugenol and methyl salicylate, possess the ability to transfer hydrogen atoms, thereby effectively eliminating radicals. Owing to their stability, the resultant phenoxyl radicals do not perpetuate the radical chain reaction but rather remain until a second radical appears, which they promptly neutralise through a radical-radical reaction^72^. (2) Terpenoids featuring a cyclohexadiene structure, such as cadina-1(6),4-diene, and cadina-1,4-diene, undergo autoxidation characterised by an exceptionally rapid termination process. It will cause an overall increase in the rate of oxidative chain termination, thereby shortening the chain length and reducing the overall rate of oxidation, as assessed by the rate of oxygen consumption or the rate of formation of oxidised products. Mechanically, they could be defined as termination-enhancing antioxidants to distinguish them from chain-breaking antioxidants^73^. These two kinds of molecules mainly contributed to the antioxidant activity of *L. bicolour* EO, while synergistic or antagonistic behaviour within EO’s molecules also holds a considerable role in overall antioxidant performance.

### Anti-acetylcholinesterase activity analysis

**Figure 4.**
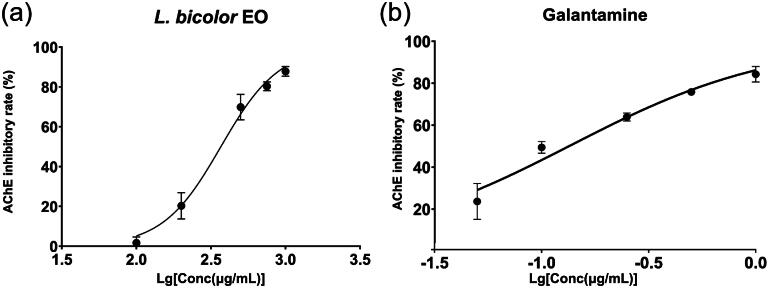
Variation in AChE inhibitory rate (%) with varying concentrations for *L. bicolour* EO (a), Galantamine (b).

*L. bicolour* EO was found to exhibit an AChE inhibition effect with an IC_50_ value of 309.30 ± 11.16 μg/mL, which is higher than the IC_50_ value of positive control galantamine (0.13 ± 0.02 μg/mL). The dose-dependent sigmoid curves of *L. bicolour* EO and galantamine are shown in Figure 4. The AChE inhibitory effect of EO may be ascribed to the monoterpenes. These secondary metabolites typically possess potent but reversible acetylcholinesterase inhibitory capacities^74^. Compared to previous EO studies, The results obtained in this study are stronger than *Ocimum americanum* (IC_50_ = 570 μg/mL) but lower than *O. basilicum* (IC_50_ = 220 μg/mL) and *O. africanum* (IC_50_ = 175 μg/mL)^75^.

**Figure 5.**
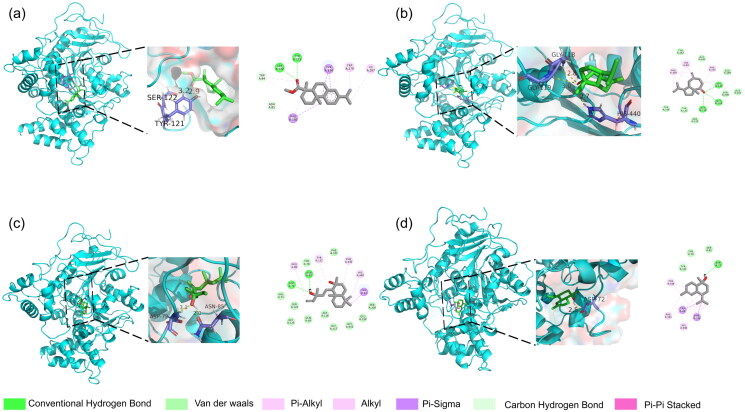
Docking interactions between selected ligands and AChE. (a) Methyl dehydroabietate; (b) Mintketone; (c) Sclareol; (d) T-Muurolol. From left to right, overall 3D, partial 3D, and 2D images in order. In 3D images, cyan cartoons for AChE protein, green sticks for small molecules, and purple sticks for amino acid residue bonded with small molecules. In 2D images, green dashed lines for hydrogen bonding, orange dashed lines for electrostatic interactions, and pink dashed lines for hydrophobic interactions.

In recent research, molecular docking has become essential for elucidating the binding affinities of bioactive compounds with target enzymes and validating their proposed mechanism of bioactivities^76^. To further identify which components dominate the anti-acetylcholinesterase activity of *L. bicolour* EO, all identified components were docked with AChE as ligands. The molecular docking results between ligands and AChE showed all ligands possessing negative binding energy values ranging from −5.1 kcal/mol to −10.1 kcal/mol, indicating favourable interactions between ligands and AChE (Supplementary Table 1). Detailed binding energy data showed that every ligand docked binding energies of 20 modes with AChE (Supplementary Table 2). Four significant docking results were visualised (Figure 5, Supplementary Figure 1), with methyl dehydroabietate forming hydrogen bonds with TYR A:121, and SER A:122, mintketone forming hydrogen bonding with GLY A:118, GLY A:119, and HIS A:440, sclareol forming hydrogen bonds with ASN A:85 and ASP A:72, while T-Muurolol bonding to ASP A:72. Hydrogen bond is an important factor that influences protein-ligand complex stability^77^. Hydrogen bonds can enhance the stability and longevity of molecules docked in an enzyme-active pocket. Previous studies have shown that sclareol has been demonstrated to exhibit significant inhibition against AChE^78^, suggesting its decisive effect on the anti-acetylcholinesterase activity of *L. bicolour* EO. Compared with major compounds of *L. bicolour* EO (β-pinene, β-phellandrene, caryophyllene, palmitinic acid), sclareol can form multiple hydrogen bonds with AChE. In contrast, the primary components of *L. bicolour* do not possess configurations that facilitate hydrogen bonding. The long-chain fatty acids (such as palmitinic acid, accounting for 5.49%) present in the EO have numerous rotational bonds, which may hinder their docking efficiency compared to sclareol. However, the limited presence of sclareol in EO diminishes its efficacy. For methyl dehydroabietate, no related research focuses on its anti-acetylcholinesterase activity. Compared with galantamine, positive control of anti-AChE experiment, and also a well-studied natural inhibitor for AChE, the binding energy of methyl dehydroabietate (−10.1 kcal/mol) is slightly lower than galantamine (−9.2 kcal/mol)^79^. Methyl dehydroabietate established hydrogen bonds and π-π stacking interactions with AChE. This phenomenon is also observed in the interrelation between galantamine and AChE. These interactions are potentially attributable to the carboxyl and aromatic ring structures, which bestow structural advantages upon methyl dehydroabietate. Therefore, further studies on developing AChE inhibitors could consider methyl dehydroabietate as a potential candidate.

### Anti-α-glucosidase activity analysis

**Figure 6.**
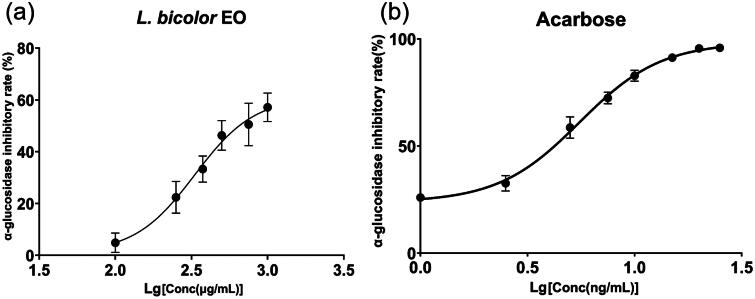
Variation in α-glucosidase inhibitory rate (%) with varying concentrations for *L. bicolour* EO (a), Acarbose (b).

α-Glucosidase, a pivotal enzyme in digestion, plays a critical role by catalysing the hydrolysis of α (1 → 4) glucosidic bonds, thereby breaking down non-absorbable complex carbohydrates into absorbable monosaccharides. This process is essential; however, excessive activity can lead to elevated blood sugar levels or hyperglycaemia. Currently, the most prominent agents for controlling hyperglycaemia are α-glucosidase inhibitors, which slow down α-glucosidase activity, effectively reducing carbohydrate hydrolysis and glucose absorption at the brush border of the small intestine. By diminishing postprandial hyperglycaemia, these inhibitors contribute to alleviating the symptoms of diabetes^80^. Therefore, searching for α-glucosidase inhibitors is essential for developing new antidiabetic drugs. In our study, the anti-α-glucosidase activity of *L. bicolour* EO was investigated. As shown in Figure 6, the IC_50_ value of *L. bicolour* EO was 360.47 ± 35.67 μg/mL, while positive control acarbose was 5.52 ± 0.22 ng/mL. Compared to *Dalea foliolosa* (IC_50_ = 133.6 ± 2.3 μg/mL)^81^, the α-glucosidase inhibitory effect of EO of *L. bicolour* is relatively lower, showing our present EO exhibits anti-α-glucosidase activity.

**Figure 7.**
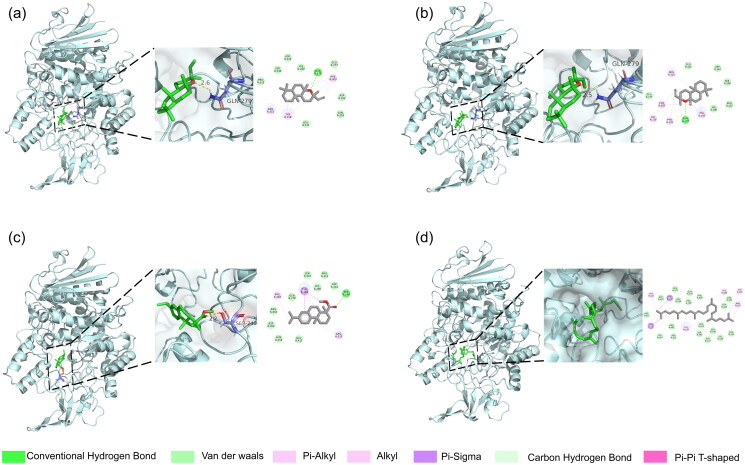
Docking interactions between selected ligands and α-glucosidase. (a) Epimanoyl oxide; (b) Manoyl oxide; (c) Methyl dehydroabietate; (d) Squalene. From left to right, overall 3D, partial 3D, and 2D images in order. In 3D images, palecyan cartoons for α-glucosidase protein, green sticks for small molecules, and purple sticks for amino acid residue bonded with small molecules. In 2D images, green dashed lines for hydrogen bonding, orange dashed lines for electrostatic interactions, and pink dashed lines for hydrophobic interactions.

To further explain the mechanism of anti-α-glucosidase activity from *L. bicolour* EO, we evaluated the interaction between identified compounds and the catalytic site of α-glucosidase by molecular docking. The binding energies of all compounds docked with α-glucosidase in the essential oil ranged from −5.1 kcal/mol to −8.6 kcal/mol (Supplementary Table 3). Detailed molecular docking data of α-glucosidase showed binding energy data of 20 modes that every ligand docked with α-glucosidase (Supplementary Table 4). Among these compounds, Methyl dehydroabietate exhibited the lowest binding energy (−8.6 kcal/mol), followed by squalene (−8.5 kcal/mol), manoyl oxide (−8.5 kcal/mol), and epimanoyl oxide (−8.5 kcal/mol). Generally, their stability in the active site is mainly attributed to electrostatic interactions, van der Waals forces, and hydrogen bonds^82^. These well-docked results were visualised (Figure 7, Supplementary Figure 2). Methyl dehydroabietate with the binding energy of −8.6 kcal/mol occupied the active site by interacting with TYR A: 158, SER A:240, PHE A:303, and ARG A:315. The oxygen of the ester group of methyl dehydroabietate formed a hydrogen bond with SER A:240 with a bond length of 2.2 Å. This hydrogen bond contributed −8.6 kcal/mol to the binding energy and can be considered a pivotal anchor for facilitating the binding of methyl dehydroabietate in the active site. Besides, the π-π T-shaped orientation was established between the benzene ring of methyl dehydroabietate and TYR A: 158, further enhancing the interaction. Manoyl oxide and epimanoyl oxide formed hydrogen bonds with GLN A:279 through their oxygen in the ether bond, with bond lengths of 2.5 Å and 2.6 Å, respectively. However, squalene mainly binds through electrostatic interactions. It is worth noting that the major components β-pinene (15.41%), β-phellandrene (12.43%), and caryophyllene (7.79%) were found to bind normally to α-glucosidase without hydrogen bonds. Methyl dehydroabietate, manoyl oxide, and epimanoyl oxide docked with α-glucosidase better may be attributed to their configurations. Compared with acarbose, positive control in the anti-α-glucosidase test, these molecules possess fewer rotatable bonds and lower polarity. Hence, these molecules exhibit lower binding energies than acarbose (−6.3 kcal/mol)^83^, which can be accounted for. Methyl dehydroabietate exhibited carboxyl and aromatic rings that formed hydrogen bonds and π-π T-shaped orientation within the active site of α-glucosidase. Their configurations are highly similar for manoyl oxide and epimanoyl oxide, and both form hydrogen bonds with α-glucosidase, illustrating their structural advantages. However, the major components in *L. bicolour* EO lacked these configurations. Therefore, the anti-α-glucosidase activity of *L. bicolour* EO may be attributed to those well-docked small molecules in low relative content. The anti-α-glucosidase activity of *L. bicolour* EO is ordinary and can be partially explained on this basis.

### Anti-β-lactamase activity analysis

**Figure 8.**
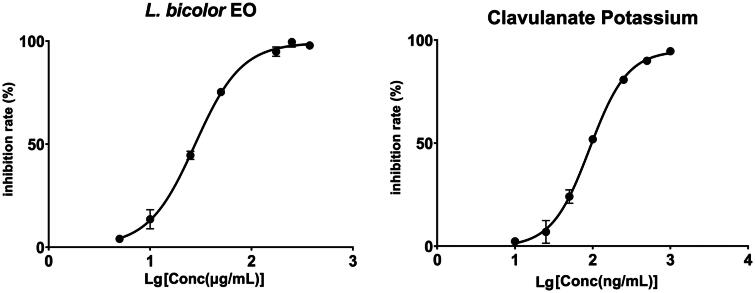
Variation in β-lactamase inhibitory rate (%) with varying concentrations for *L. bicolour* EO (a), Clavulanate Potassium (b).

Due to the long-term use of antibiotics, bacterial resistance has become an issue demanding prompt solution^14^. Among the various mechanisms of bacterial resistance, the expression of β-lactamase enzymes has received significant attention and has become a subject of extensive research. As shown in Figure 8, the anti-β-lactamase activity of *L. bicolour* EO was characterised by an IC_50_ value of 27.54 ± 1.21 μg/mL. The IC_50_ value of positive control, clavulanate potassium, was 91.02 ± 3.47 ng/mL. The IC_50_ of *L. bicolour* EO was slightly higher compared to methyl cinnamate isolated from *O. basilicum* essential oil (IC_50_ = 11.6 µg/mL)^43^, indicating *L. bicolour* EO exhibits intense anti-β-lactamase activity and the synergistic antimicrobial potential.

**Figure 9.**
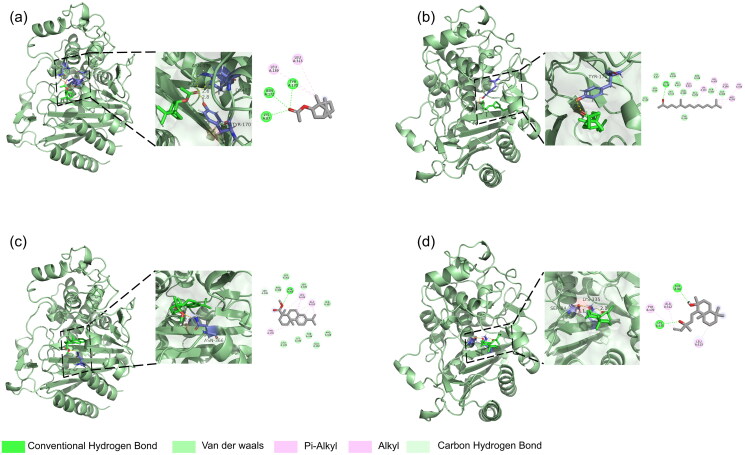
Docking interactions between selected ligands and β-lactamase. (a) (+)-Borneol acetate; (b) Farnesyl acetone; (c) Methyl dehydroabietate; (d) Sclareol. From left to right, overall 3D, partial 3D, and 2D images in order. In 3D images, palegreen cartoons for β-lactamase protein, green sticks for small molecules, and purple sticks for amino acid residue bonded with small molecules. In 2D images, green dashed lines for hydrogen bonding, orange dashed lines for electrostatic interactions, and pink dashed lines for hydrophobic interactions.

Molecular docking was performed to further understand the interaction between the components of *L. bicolour* EO and the β-lactamase enzyme. The binding energies of each component docked with β-lactamase ranged from −4.2 kcal/mol to −7.0 kcal/mol (Supplementary Table 5). Detailed molecular docking data showed that every ligand docked binding energies of 20 modes with β-lactamase (Supplementary Table 6). Four well-docked results were selected to be visualised (Figure 9, Supplementary Figure 3). (+)-Borneol acetate formed hydrogen bonds with LYS A: 87, TYR A: 170, and ASN A:172, with bond distances of 2.6 Å, 2.8 Å, and 2.7 Å, respectively. These hydrogen bonds stabilised the conformation of (+)-borneol acetate in the tested active pocket, showing its potential as a β-lactamase inhibitor. Farnesyl acetone formed a hydrogen bond with TYR A:170 with distance of 2.8 Å, while sclareol formed two hydrogen bonds with SER A: 84 and LYS A:335, with distances of 2.1 Å and 2.1 Å, respectively. Interestingly, the main component identified in *L. bicolour* EO, β-pinene, exhibited the highest binding energy (−4.2 kcal/mol), indicating it may not be the main factor contributing to the strong anti-β-lactamase activity of *L. bicolour* EO. The rationale behind this is that (+)-borneol acetate is capable of forming multiple hydrogen bonds with β-lactamase, whereas β-pinene lacks the structural configuration that facilitates hydrogen bonding, potentially impeding its docking efficiency in comparison to (+)-borneol acetate.

## Conclusion

In the present study, the chemical composition, antioxidant, anti-acetylcholinesterase, anti-α-glucosidase, and anti-β-lactamase activities of the EO from *L. bicolour* were investigated for the first time. Eighty-three compounds were identified, with the main components of β-pinene, β-phellandrene, and caryophyllene. The *L. bicolour* EO exhibited strong *in vitro* antioxidant activity, anti-β-lactamase activity, AChE, and α-glucosidase inhibitory effect. These *in vitro* tests provide preliminary data on biological activities. These biological activities show that *L. bicolour* EO exhibits translational potential in pharmaceuticals and agriculture. However, the *in vivo* situation is much more complex than *in vitro* experiments. Future *in vivo* research needs to consider the potential side effects of the essential oil within the body and assess whether it can effectively reach its target sites. Other components in the body may also interact with essential oils. Factors such as absorption, half-life, metabolism, and elimination must also be considered. Therefore, *in vivo* studies coupled with pharmacodynamics and pharmacokinetics are essential for further developing and applying *L. bicolour* EO. Molecular docking was conducted to identify the components from *L. bicolour* EO with the most potent inhibitory effects on the tested enzymes. The results showed that methyl dehydroabietate was the major active composition that inhibited all the tested enzymes. Sclareol and (+)-borneol acetate showed strong binding affinity to α-glucosidase and β-lactamase, respectively. This study fills the gap in bioactive compounds of *L. bicolour* EO and provides a direction for searching enzyme inhibitors for these three tested enzymes.

## Supplementary Material

Supplementary Figure 3.tif

Supplementary Figure 2.tif

Supplementary Table 3 The minimum binding energy of each EO component with α glucosidase.xlsx

Supplementary Table 4 Detailed molecular Docking Data of α glucosidase.xlsx

Supplementary Figure 1.tif

Supplementary Table 5 The minimum binding energy of each EO component with β lactamase.xlsx

Supplementary Table 1 The minimum binding energy of each EO component with AChE.xlsx

Supplementary Table 2 Detailed molecular Docking Data of AChE.xlsx

Supplementary Table 6 Detailed molecular Docking Data of β lactamase.xlsx

## Data Availability

Data is available on request from the authors.
