# Supplementary figures and images for: Chemical composition, antioxidant activities, and enzyme inhibitory effects of Lespedeza bicolour Turcz. essential oil
Source: J Enzyme Inhib Med Chem. 2025 Feb 6;40(1):2460053. doi: 10.1080/14756366.2025.2460053 (PMC11803819; doi:10.1080/14756366.2025.2460053)

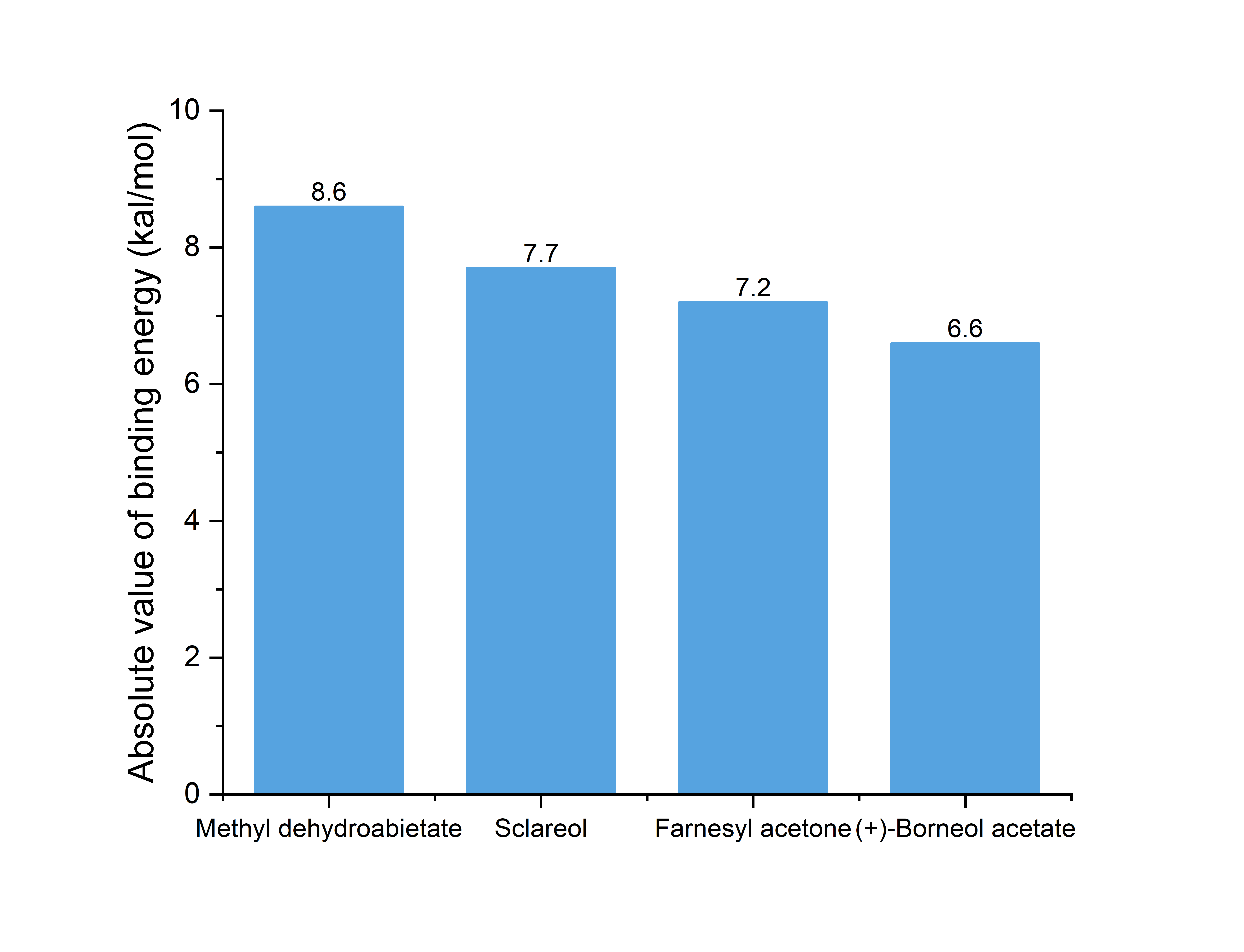

Supplement: Supplementary Figure 3.tif [file IENZ_A_2460053_SM1965.tif]

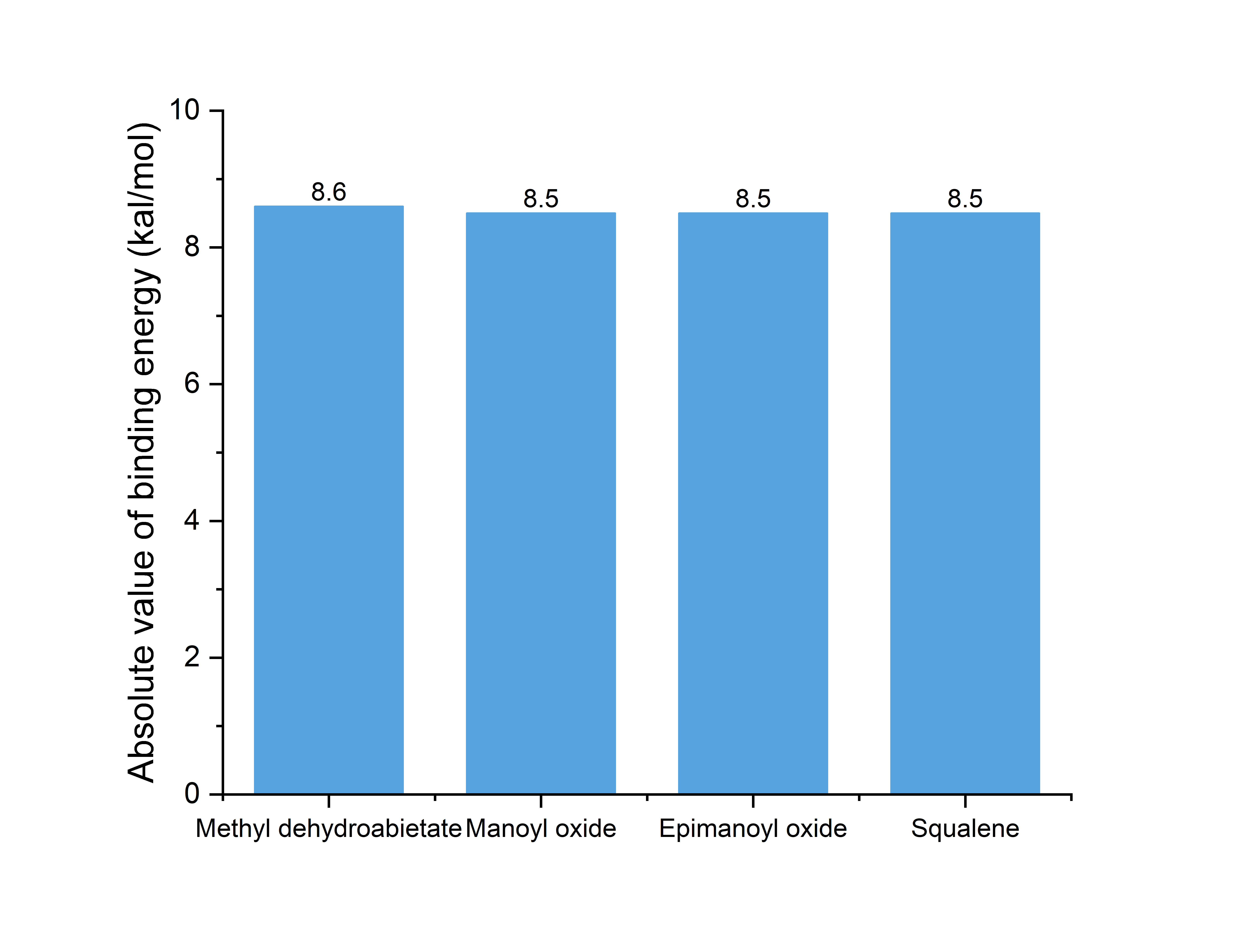

Supplement: Supplementary Figure 2.tif [file IENZ_A_2460053_SM1964.tif]

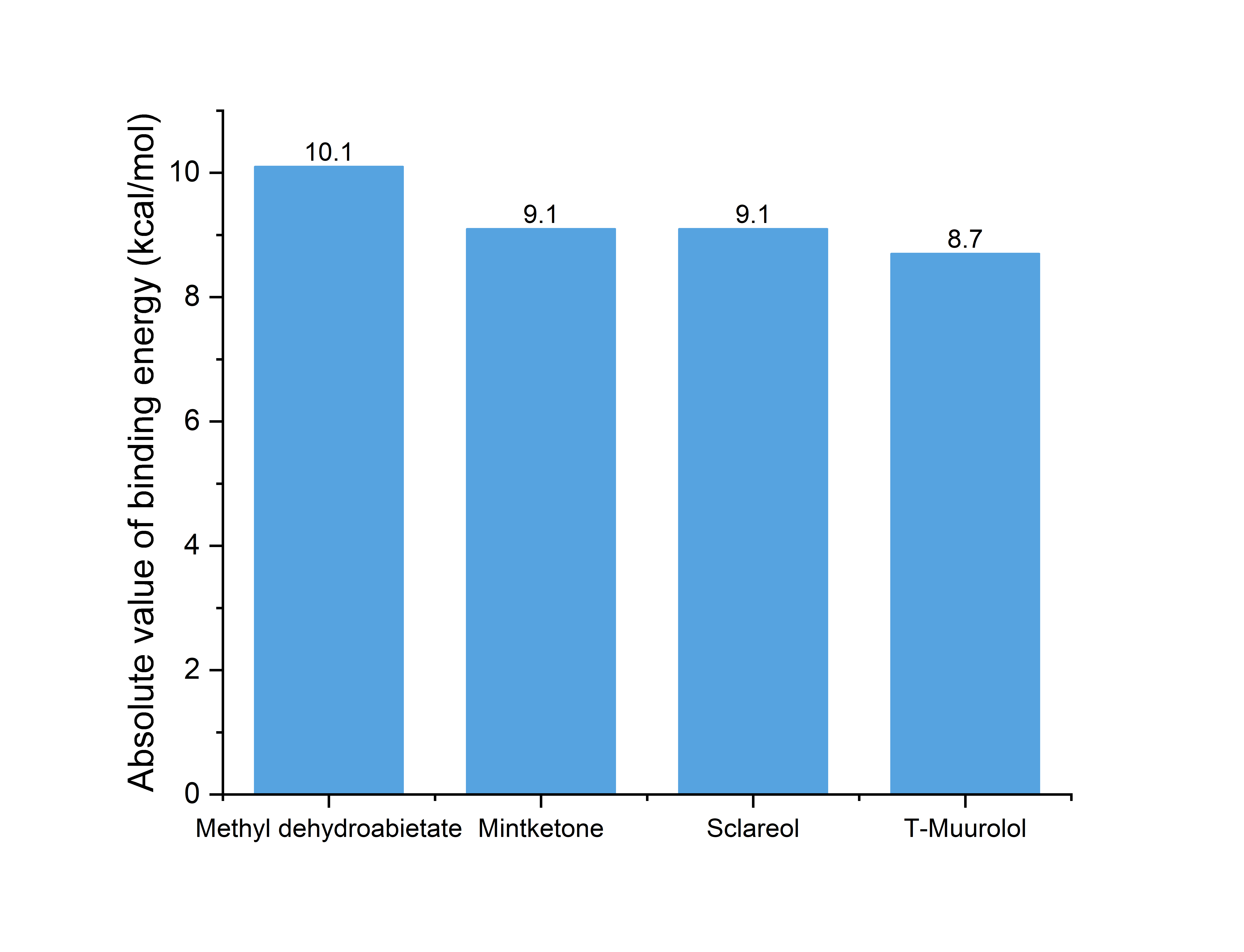

Supplement: Supplementary Figure 1.tif [file IENZ_A_2460053_SM1961.tif]
